# Adaptive Evolution of GatC, a Component of the Galactitol Phosphotransferase System, for Glucose Transport in *Escherichia coli*

**DOI:** 10.4014/jmb.2502.02021

**Published:** 2025-04-23

**Authors:** Su On Jeong, Hyun Ju Kim, Sang Jun Lee

**Affiliations:** 1 Department of Systems Biotechnology, and Institute of Microbiomics, Chung-Ang University, Anseong 17546, Republic of Korea; 2 Biological Resources Research Department, Nakdonggang National Institute of Biological Resources (NNIBR), Sangju 37242, Republic of Korea

**Keywords:** CRISPR-Cas9, *gatC*, glucose transport, fermentation, metabolic adaptation, phosphotranferase system (PTS)

## Abstract

Microbial adaptive laboratory evolution is a powerful approach for uncovering novel gene functions within metabolic pathways. Building on our previous discovery of ExuT as a glucose transporter in *ptsG*-deficient *Escherichia coli*, this study investigates strains lacking recognized glucose transporters (*ptsG*, *manX*, and *exuT*). Successive rounds of experimental evolution revealed key genetic adaptations, including loss-of-function mutations in *malI* and *nagC*, which encode repressors of the maltose and N-acetylglucosamine phosphotransferase systems (PTS), respectively. Additionally, a gain-of-function mutation in *gatC*, a component of the galactitol PTS EIIC, was identified. The functional significance of these mutations was validated through transcript analysis, genetic knockouts, and CRISPR-Cas9-mediated site-specific genome mutagenesis, with a particular focus on the *gatC* missense mutation (F340C). The resulting modifications were examined for their effects on sugar specificity and metabolic flux. Furthermore, our findings identified succinate as the predominant fermentation product in engineered strains utilizing alternative glucose transport pathways, including the maltose, N-acetylglucosamine, and galactitol PTS. This study advances our understanding of sugar transport mechanisms in *E. coli* and offers insights into regulatory networks, fermentative metabolism, and substrate specificity, which can be leveraged for evolutionary engineering in biotechnological applications.

## Introduction

Central metabolism is essential for the survival of bacteria because it provides both energy and biosynthetic precursors [1]. While most animals utilize only a few monosaccharides (such as glucose, galactose, and fructose), bacteria possess numerous sugar-specific transporters for mono-, di-, and trisaccharides [2, 3]. These bacterial sugar transporters are classified into three groups based on their energy source: (i) the phosphotransferase system (PTS), which utilizes phosphoenolpyruvate (PEP) for group translocation; (ii) ATP-binding cassette (ABC) transporters, which depend on ATP hydrolysis; and (iii) secondary active transporters, such as the major facilitator superfamily (MFS), which rely on ion gradients [4].

Sugars often have multiple transport pathways, with some transporters capable of recognizing and importing various sugars. For example, galactose transport in *E. coli* occurs through GalP (a galactose:H^+^ symporter) and MglBAC (a galactose-specific ABC transporter) [5], while EIICD^Man^ (the mannose PTS) transports both mannose and glucose [6]. Glucose is one of the preferred carbon and energy sources in many bacteria, and the transcriptional regulation mechanism known as carbon catabolite repression (CCR), along with glucose transport through glucose-specific PTS (EIICB^Glc^), has been widely studied [7].

In the absence of glucose-specific PTS, alternative glucose transporters can facilitate uptake, with their expression regulated by factors such as carbon source availability, oxygen conditions (aerobic or anaerobic), and the presence of competing sugars [8]. In *E. coli*, GalP, MglBAC, and EIIBC^Mal^ (the maltose PTS) are known to serve as alternative glucose transport systems [9].

Studies have shown that mutations acquired during adaptive evolution can alter sugar specificity in transporters. For instance, in the JU15 strain, a mutation in GatC (GatC^S184L^), a component of the galactitol PTS, enhanced lactate productivity and xylose consumption [10]. Similarly, mutations in the arabinose-proton symporter (AraE^D223Y^) and the regulatory protein of the arabinose operon (AraC^L156I^) enabled xylose transport [11]. Another study demonstrated that the NagE^M263K^ mutation facilitated glucose transport in a strain lacking glucose-specific transporters [12].

Previously, through adaptive evolution in an *E. coli* strain lacking the glucose PTS genes *ptsG* and *manX*, we identified ExuT as a novel glucose transporter that redirected metabolic flux toward succinate production during fermentation [13]. Additionally, we found that *E. coli* strain C, depending on its genetic background, can transport glucose via the N-acetylgalactosamine PTS [14].

In this study, we subjected *E. coli* strains lacking known glucose transporters (Δ*ptsG* Δ*manX* Δ*exuTR* or Δ*ptsGHI* Δ*exuTR*) to adaptive evolution to identify novel glucose uptake mechanisms. We improved the growth rate and glucose transport efficiency of the evolved strains and validated their genetic modifications through whole-genome sequencing. Furthermore, we characterized these strains by analyzing gene expression and fermentation profiles, uncovering mechanisms of anaerobic glucose transport and associated metabolic pathways. These findings expand our understanding of bacterial glucose transport and metabolic adaptation, with potential applications in industrial biotechnology.

## Material and Methods

### Bacterial Strains

The *E. coli* strains and plasmids used in this study are listed in Table S1. The *E. coli* K-12 BW25113 strain was obtained from the Coli Genetic Stock Center (CGSC) at Yale University, and the P1 *vir* phage was generously provided by Dr. Sankar Adhya at the National Institutes of Health (NIH). The primers used in this study are listed in Table S2.

### Chromosome Manipulation

The Keio collection, purchased from Open Biosystems (USA), was used to generate mutant *E. coli* strains. In this collection, single-gene open reading frames (ORFs) were replaced with kanamycin resistance (Km^R^) cassettes, which served as templates for introducing mutations [15]. Mutations were introduced via standard P1 transduction and λ Red recombineering system. Kanamycin-sensitive strains were generated by introducing the pCP20 plasmid, which removed the Km^R^ cassette via FLP recombinase [16, 17]. Detailed strain construction methods are described in the Supplementary Data.

### Anaerobic Fermentation

LB broth (Cat. No. LB-05) was purchased from LPS Solution (Republic of Korea), while yeast extract (Cat. No. 9235Y) was purchased from GenomicBase (Republic of Korea). Additional reagents were purchased as follows: sodium bicarbonate (Cat. No. S6014), sodium phosphate monobasic monohydrate (Cat. No. S9638), potassium phosphate dibasic (Cat. No. P3786), and sodium sulfide nonahydrate (Cat. No. 431648) from Sigma-Aldrich (USA. No. 64220-0601) from Junsei Chemical Co., Ltd. (Japan); and galactitol (Cat. No. G0005) from Tokyo Chemical Industry Co. Ltd., (Japan).

Bacterial starter cultures were grown in 5 ml of LB broth at 37°C with shaking (180 rpm). The fermentation medium (final volume: 1 L) contained 5 g yeast extract, 9 g D-glucose or galactitol (final concentration: 50 mM), 10 g sodium bicarbonate, 8.5 g sodium phosphate monobasic monohydrate, and 15.5 g potassium phosphate dibasic. Sodium sulfide (1 mM) was added to remove dissolved oxygen, creating strictly anaerobic conditions. Fermentations were performed in 125 ml serum vials containing 100 ml of medium, inoculated with 1% (v/v) seed culture. The vial headspace was flushed with nitrogen gas, and cultures were incubated anaerobically at 37°C with shaking (180 rpm).

### Analytical Procedures

Bacterial growth was monitored by measuring the optical density at 600 nm (OD_600_) using a Biochrom Libra S70 Double Beam Spectrophotometer (Biochrom Ltd., UK). Cell cultures were diluted (1:10) in PBS before OD_600_ measurements.

Metabolite concentrations (D-glucose, succinate, lactate, formate, acetate, and ethanol) were determined via high-performance liquid chromatography (HPLC). Briefly, culture supernatants were obtained by centrifugation (12,000 rpm, 4°C, 10 min) and filtered through Nylon Syringe Filter (0.22 μm pore size). Analysis was performed on an Agilent 1100 Series HPLC system equipped with an Aminex HPX-87H column (300 × 7.8 mm; Bio-Rad Laboratories, Inc., USA). The column temperature was maintained at 47°C, and a 0.01 N H_2_SO_4_ solution served as the mobile phase at a flow rate of 0.5 ml min^–1^.

### Genome Analysis

Genomic DNA was extracted using the Wizard Genomic DNA purification kit (Cat. No. A1120; Promega, USA). Whole-genome sequence analysis was conducted by Ebiogen Inc. (Republic of Korea). Libraries were prepared using the TruSeq Nano DNA High Throughput Library Prep Kit (Illumina) following the manufacturer’s guidelines. Details on whole-genome sequencing and variant calling methods are provided in the Supplementary Data. Sequencing data are available in the NCBI BioProject (PRJNA1053494) under accession numbers SRR27228827, SRR27228826, SRR27228825, SRR27228823, and SRR27228824.

### Transcript Analysis

Transcription levels of *malXY*, *nagE*, and *nagB* were quantified using RT-qPCR. Cells were grown anaerobically in a fermentation medium at 37°C with shaking (180 rpm). After 6 h of incubation, cells were harvested, and total RNA was extracted using the RNeasy Mini Kit (Cat. No. 74104; Qiagen, Germany).

Primers were designed using the Eurofins Genomics qPCR Assay Design Tool (https://eurofinsgenomics.eu/en/ecom/tools/qpcr-assay-design/). RT-qPCR was performed on a CFX Connect system (Bio-Rad, USA) using the RealHelix qPCR Kit (Cat. No. QRT-S500; NanoHelix, Republic of Korea). The reaction was performed using 5 ng of total RNA under the following conditions: reverse transcription at 50°C for 40 min, followed by initial denaturation at 95°C for 12 min and 40 cycles of PCR amplification, consisting of denaturation at 95°C for 20 s and annealing and extension at 60°C for 1 min. Raw fluorescence data were normalized using the expression levels of 16S ribosomal RNA and target genes in wild-type BW25113 cells.

### Genome Editing

To generate Cas9-harboring strains, P1 *vir* phage lysates containing the *araBAD*::*P_BAD_*-*cas9*-Km^R^ cassette from HK1059 [18] were used to transduce the HK1319 and SO039 strains, resulting in the SO068 and SO069 strains, respectively.

The sgRNA expression plasmid was constructed by designing an sgRNA containing a target recognition sequences (TRSs) at position T1019, corresponding to the mutation site in the *gatC* gene. The spectinomycin-resistance gene and sgRNA sequence were amplified from the pHL003 template, omitting the ampicillin-resistance gene. The two PCR fragments were then purified and assembled using the Gibson Assembly Master Mix (Cat. No. E2611; NEB, England). Mutagenic oligonucleotides were designed (as detailed in Table S2) to introduce a random codon at the target site.

*E. coli* SO068 and SO069 harboring pHK463 were cultured in LB supplemented with ampicillin (50 μg ml^-1^) at 30°C. At an OD_600_ of 0.4, L-arabinose (final concentration: 1 mM) was added to induce Cas9 and lambda Bet protein expression for oligonucleotide-directed mutagenesis. Electrocompetent *E. coli* cells were prepared as described previously [18]. For negative selection, sgRNA plasmids (200 ng) and mutagenic-oligonucleotides (100 pmol) were electroporated into pHK463-harboring *E. coli* SO068 and SO069 cells, in which Cas9 and lambda Bet proteins were fully expressed.

Electroporation was performed at 25 μF, 200 Ω, and 1.8 kV in a 0.1-cm electroporation cuvette. Immediately after, the cells were transferred to 950 μl SOC medium, incubated at 30°C with shaking (180 rpm) for 1 h, and plated on LB agar containing spectinomycin (75 μg ml^-1^). The plates were incubated for 16 h at 30°C. Transformants were streaked onto the fermentation medium agar and incubated anaerobically for 18 h at 37°C using an AnaeroGen W-Zip Compact (Cat. NO 23-313-398; Oxoid, UK). Among the genome-edited transformants, candidates with colony formation rates comparable to the adapted progeny strain HK1181 were selected. Subsequently, their *gatC* gene was PCR amplified, and mutations were confirmed through Sanger sequencing.

## Results

### Adaptation and Fermentation Profiles of Alternative Glucose Transporters

To investigate alternative glucose transport mechanisms, glucose transporter-deficient HK1161 (Δ*ptsG* Δ*manX* Δ*exuTR*), SO023 (Δ*ptsG* Δ*manX* Δ*exuTR* Δ*malIXY*), and HK1162 (Δ*ptsGHI* Δ*exuTR*) strains were subjected to anaerobic conditions with D-glucose as the sole carbon source. These strains exhibited prolonged lag phases (≥ 48 h) with minimal glucose consumption. Adapted progeny strains with improved glucose uptake and growth rates were isolated through dilution, plating on LB agar, and subsequent anaerobic selection.

**Fig. 1:**
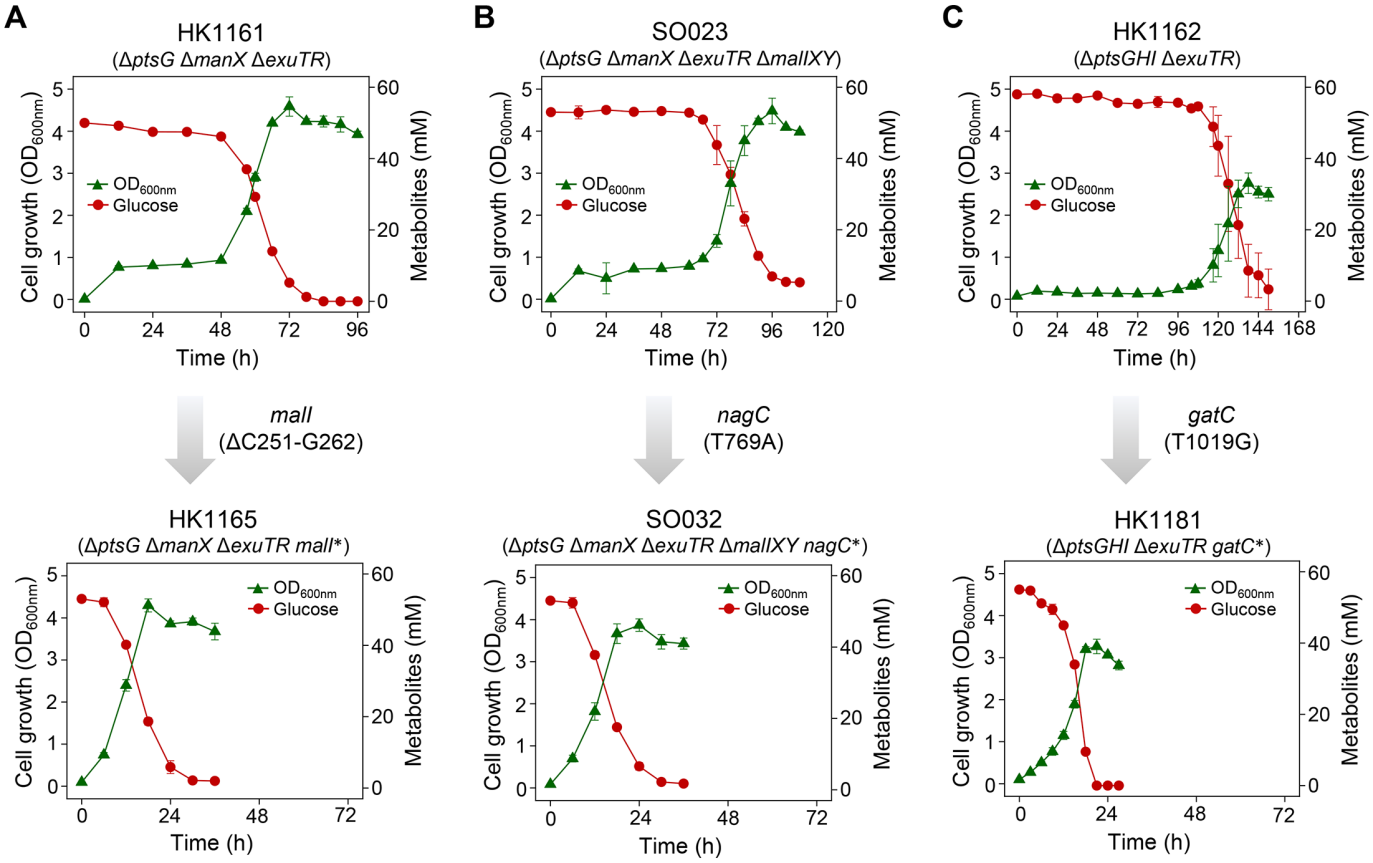
Growth and glucose consumption profiles of parental strains and adapted *Escherichia coli* strains. Each panel shows the growth and glucose consumption of a parental strain (top) and its adapted strain (bottom) with adaptive mutations acquired during anaerobic cultivation: (**A**) *malI* (ΔC251-G262), (**B**) *nagC* (T769A) and (**C**) *gatC* (T1019G). Asterisks indicate adaptive mutations.

HK1161 displayed a 48 h lag phase, reaching a peak OD_600_ of 4.6 at 72 h and consuming 50 mM glucose within 84 h. Succinate was the primary fermentation product (36.0 mM), with no detectable lactate (Fig. 1A and Table 1). SO023 displayed a 60 h lag phase, reaching a peak OD_600_ of 4.5 at 96 h and consuming nearly all glucose (leaving 5.3 mM) by 108 h, producing 33.0 mM succinate without lactate (Fig. 1B and Table 1). HK1162 exhibited the longest lag phase (108 h), reaching a peak OD_600_ of 2.76 at 138 h, which was lower than that observed in HK1161 and SO023 (Fig. 1C). By 150 h, 3.3 mM glucose remained, and ethanol was produced in minimal quantities (2.6 mM), while succinate remained the dominant product (Table 1).

The adapted progeny strains exhibited distinct fermentation profiles. HK1165 and SO032, derived from HK1161 and SO023, respectively, retained their parental fermentation characteristics, with succinate as the primary product and no detectable lactate. In contrast, HK1181, derived from HK1162, showed reduced succinate yield and increased ethanol production, distinguishing it from its parental strain (Table 1). Other progeny strains derived from HK1161 and SO023 followed similar fermentation trends (Table S3 and S4).

### Genomic Mutations in Adapted Strains

Whole-genome sequencing of adapted strains (HK1165, SO032, and HK1181) and comparison with the reference genome BW25113 (Accession No. CP009273) revealed mutations in three genes (Table 1).

HK1165, derived from HK1161 (Δ*ptsG* Δ*manX* Δ*exuTR*), carried a 12 bp deletion (ΔC251-G262) in the *malI* gene, partially deleting the maltose regulon regulatory protein (MalI) sequence. Further Sanger sequencing of *malI* in additional progeny strains (SO070–SO075) from HK1161 identified various mutations, including nucleotide substitutions, insertions, and deletions, all resulting in nonsense mutations in MalI (Table 2).

In SO032, derived from SO023 (Δ*ptsG* Δ*manX* Δ*exuTR* Δ*malIXY*), a base substitution (T769A) in the *nagC* gene encoding the N-acetylglucosamine regulon regulatory protein, was identified. Subsequent Sanger sequencing of *nagC* in strains SO034, SO035, and SO037—strains with similar growth and fermentation profiles to SO032—confirmed the presence of similar substitutions, which ultimately induced missense mutations in NagC (Table 2).

HK1181, derived from HK1162 (Δ*ptsGHI* Δ*exuTR*), exhibited a nucleotide substitution (T1019G) in *gatC*, which encodes the galactitol-specific PTS EIIC component, leading to a missense mutation in GatC (Table 2).

### Disruption of *malI* and *nagC* Genes Activates Maltose and N-Acetylglucosamine PTS

Mutations in *malI* and *nagC*, which encode transcriptional regulators for the maltose regulon and N-acetylglucosamine regulon, respectively, were identified in adapted progeny strains. Therefore, the impact of these mutations on the expression of genes regulated by each transcriptional regulator was assessed.

**Fig. 2:**
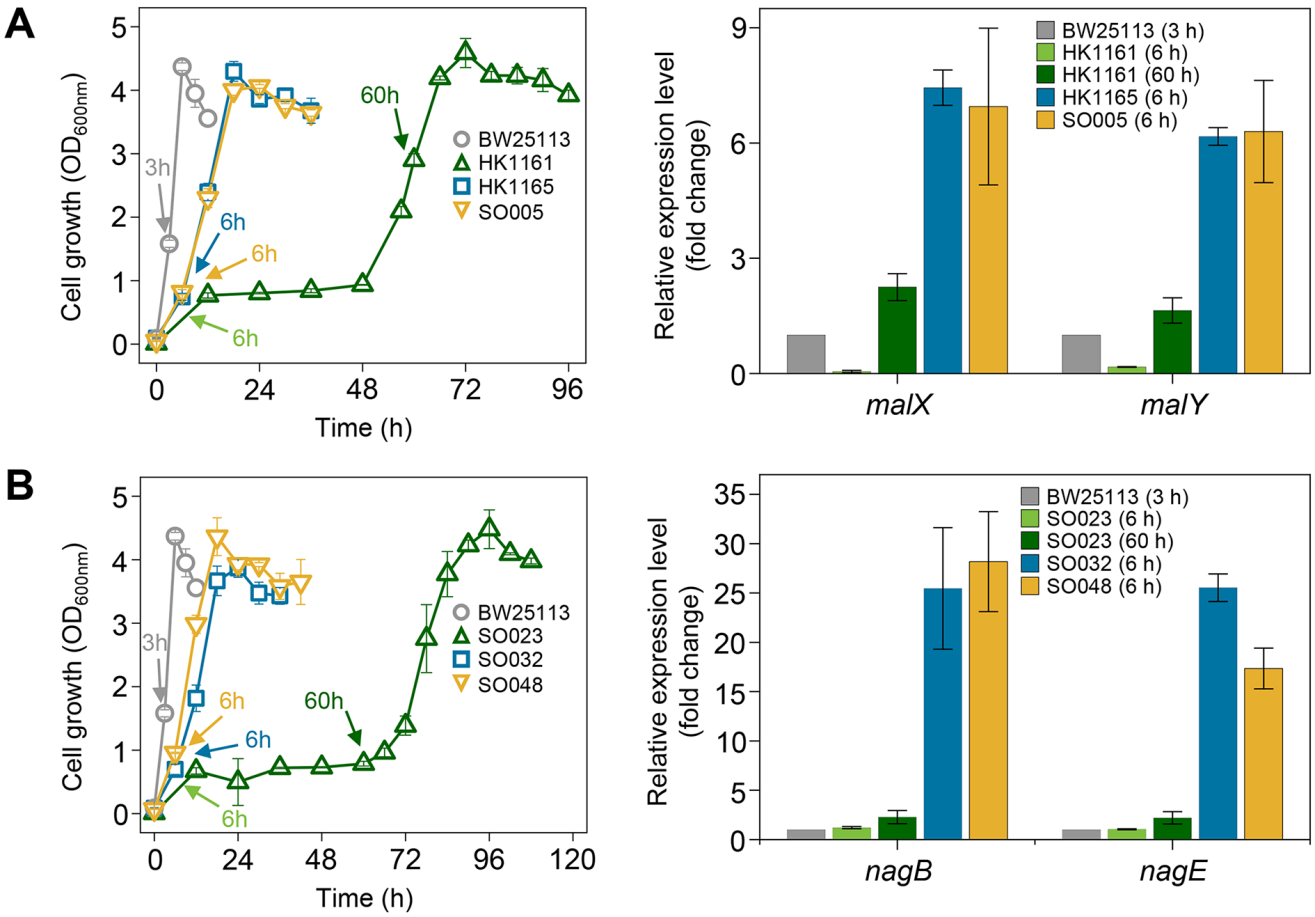
Transcript analysis of (A) maltose phosphotransferase (PTS) genes and (B) N-acetylglucosamine PTS genes of wild-type, parental, and anaerobically adapted *Escherichia coli* strains during glucose transport. RNA was isolated at the indicated sampling points (marked by arrows) and analyzed using RT-qPCR to evaluate gene expression changes associated with alternative glucose uptake.

Given the partial deletion and nonsense mutations in *malI* during adaptive evolution, these alterations likely led to a loss of MalI function. RT-qPCR analysis confirmed this by measuring the transcript levels of *malX* and malY, genes repressed by MalI. Compared to wild-type BW25113, the parental strain HK1161 (Δ*ptsG* Δ*manX* Δ*exuTR*) exhibited minimal expression of these genes during the lag phase, with a moderate increase (2.3-fold for *malX* and 1.6-fold for *malY*) at 60 h, corresponding to active cell growth. In contrast, HK1165 (carrying the *malI* ΔC251-G262 mutation in the HK1161 genetic background) showed substantially higher expression levels, with *malX* and *malY* increasing 7.4-fold and 6.2-fold, respectively, compared to the wild-type (Fig. 2A). These findings suggest that the expression of maltose transporter genes is upregulated due to the inactivation of MalI.

A missense mutation in *nagC* is expected to affect the expression of the N-acetylglucosamine PTS genes, which are repressed by NagC. RT-qPCR analysis of *nagB* and *nagE* genes, key components of this system revealed that parental strain SO023 (Δ*ptsG* Δ*manX* Δ*exuTR* Δ*malIXY*) showed transcript levels comparable to wild-type BW25113 during the lag phase (6 h), with slight increases (2.3-fold and 2.2-fold, respectively) at 60 h. However, in SO032 (carrying the *nagC* T769A mutation in the SO023 genetic background), transcript levels of *nagB* and *nagE* were significantly elevated (both 25.5-fold) (Fig. 2B). These results confirm that NagC inactivation enhances the expression of N-acetylglucosamine transporter genes.

Strains SO005 (Δ*ptsG* Δ*manX* Δ*exuTR* Δ*malI*) and SO048 (Δ*ptsG* Δ*manX* Δ*exuTR* Δ*malIXY* Δ*nagC*), derived from HK1161 and SO023 through *malI* and *nagC* deletions, respectively, exhibited transcript level increases similar to HK1165 and SO032 (Fig. 2). Additionally, Δ*ptsG* Δ*manX* Δ*exuTR* Δ*malIXY* and Δ*ptsG* Δ*manX* Δ*exuTR* Δ*malIXY* Δ*nagC* Δ*nagE* strains showed no growth until 60 h (Fig. 1B and S1), suggesting that adapted strains with disrupted *malI* and *nagC* genes transport D-glucose through maltose PTS (MalX) and N-acetyl glucosamine PTS (NagE) (Fig. S2A and S2B).

### Gain-of-Function of GatC for Glucose Transport

Unlike maltose PTS and N-acetylglucosamine PTS, which were activated by mutations in their repressors, mutations in the galactitol PTS occurred in one of its components (GatC, EIIC^Gat^). Similar to other PTSs, galactitol PTS requires the sugar non-specific proteins HPr and EI (encoded by *ptsH* and *ptsI*) for phosphorylation and transport of galactitol [19]. To determine whether the adaptive mutation in *gatC* (F340C) enabled GatC to transport glucose independently of the *ptsHI* genes, we introduced random amino acid substitutions at F340 of the *gatC* gene in two different genetic backgrounds (Δ*ptsG* Δ*manX* Δ*exuTR* Δ*malIXY* and Δ*ptsGHI* Δ*exuTR*) (Fig. S3).

**Fig. 3:**
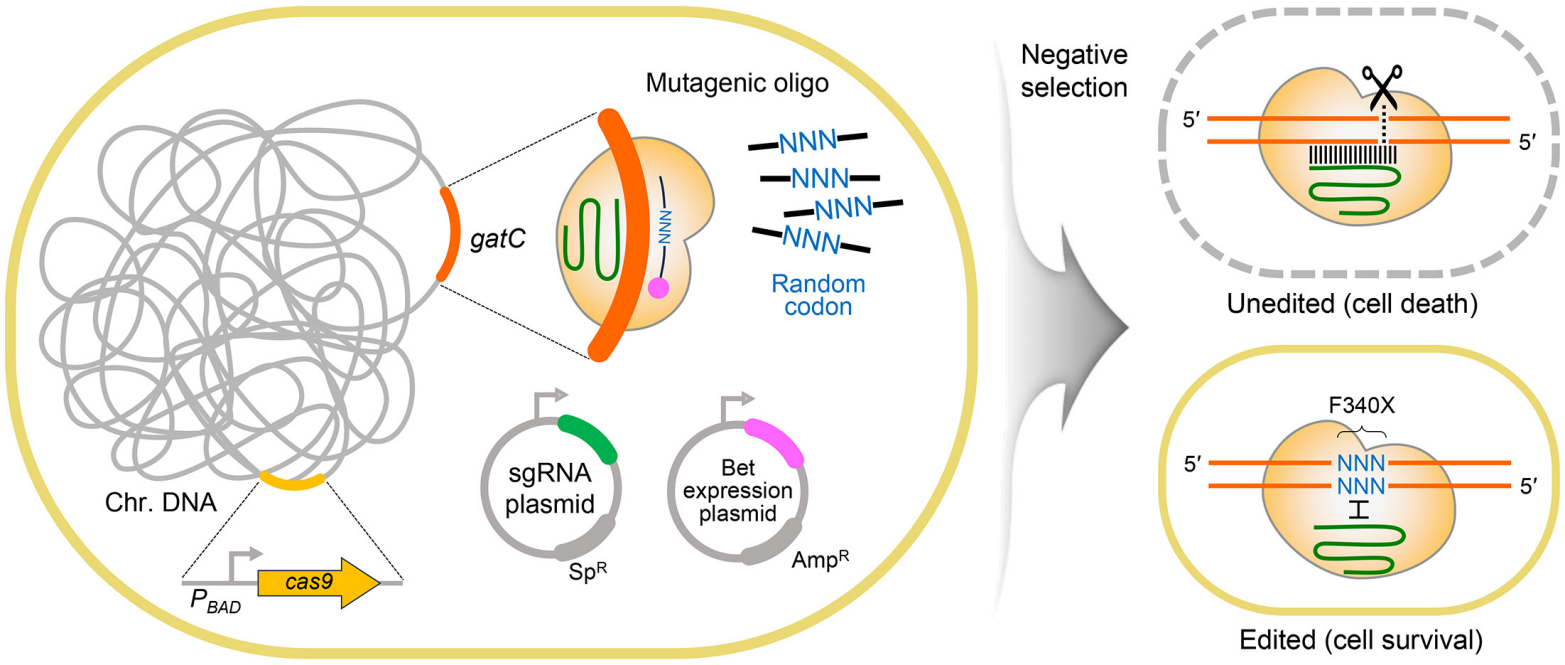
Random amino acid substitutions at position F340 of the *gatC* gene using the CRISPR-Cas9 system. Mutagenesis was performed in SO068 and SO069 strains, which carried either the Δ*ptsGHI* Δ*exuTR* or Δ*ptsG* Δ*manX* Δ*exuTR* Δ*malIXY* genotype, to introduce random amino acids at F340 in both genetic backgrounds.

CRISPR-Cas9-mediated site-directed random mutagenesis was used to generate *gatC* variants (Fig. 3). A mutagenic oligonucleotide and sgRNA were co-transformed to introduce random codons at F340 of *gatC*, and mutants were screened. Colony-forming unit (CFU) counts decreased due to negative selection by sgRNA and CRISPR-Cas9 at the target site (Fig. S3A). Selected mutants were cultured anaerobically on fermentation medium agar to select fast-growing transformants, followed by colony purification on LB medium. Sanger sequencing confirmed the *gatC* mutations, and the selected strains were further analyzed for fermentation metabolite production.

**Fig. 4:**
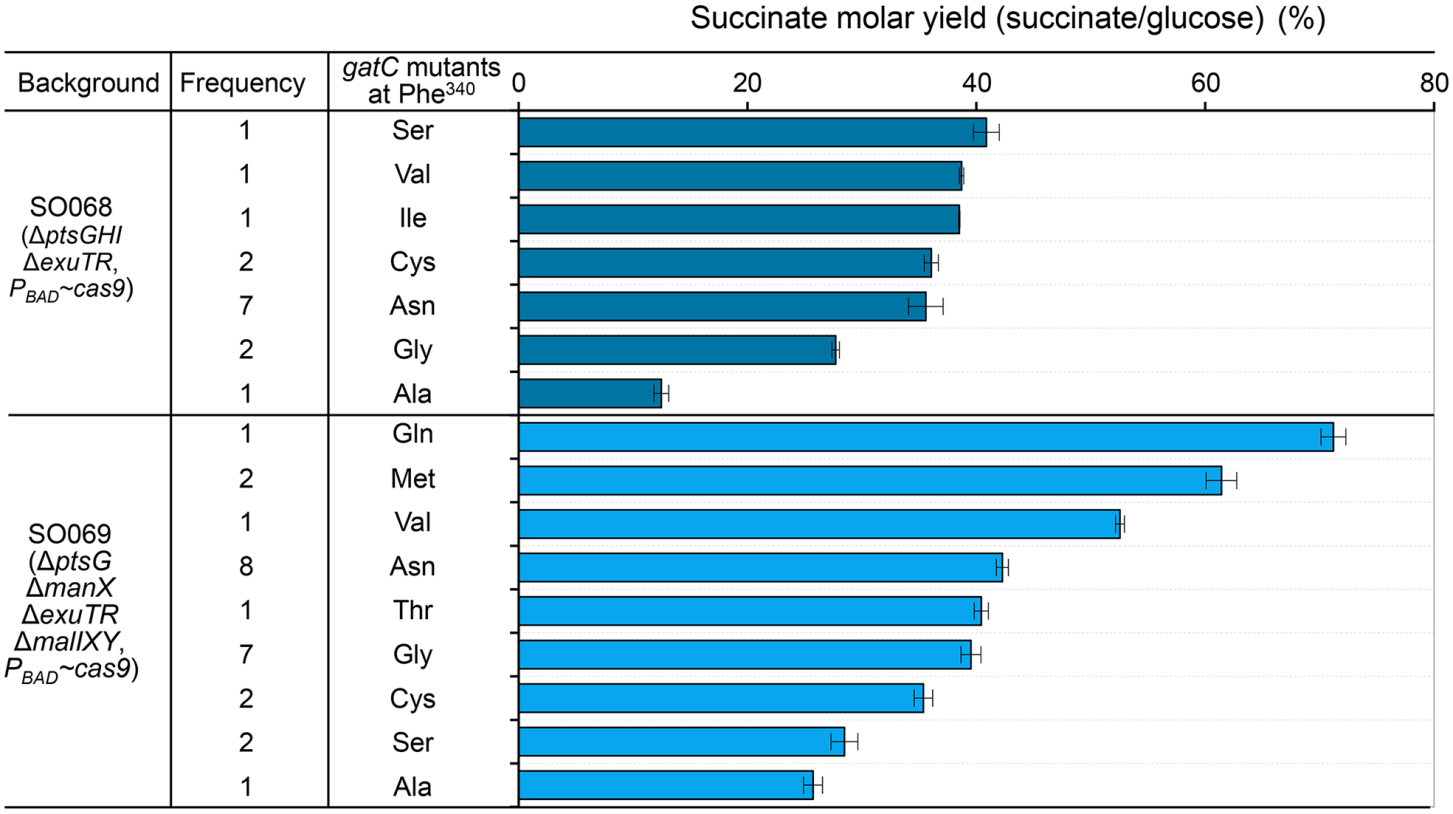
Succinate yield in mutant strains generated through CRISPR-Cas9-mediated mutagenesis. Random codons were introduced at position Phe^340^ of the *gatC* gene in the SO068 (Δ*ptsGHI* Δ*exuTR*, *P_BAD_*~*cas9*) and SO069 (Δ*ptsG* Δ*manX* Δ*exuTR* Δ*malIXY*, *P_BAD_*~*cas9*) strains.

From these selections, 13 and 25 candidates were obtained in the SO068 (Δ*ptsGHI* Δ*exuTR*, *P_BAD_*~*cas9*) and SO069 (Δ*ptsG* Δ*manX*, Δ*exuTR* Δ*malIXY*, *P_BAD_*~*cas9*) backgrounds, respectively (Fig. S3B and S3C). The *gatC* sequences of the final selected mutants were analyzed by Sanger sequencing, revealing amino acid substitutions at residue 340 of GatC. SO068 exhibited substitutions with Ala, Arg, Asn, Cys, Gly, Ile, Ser, Trp, and Val, while SO069 exhibited substitutions with Ala, Asn, Cys, Gln, Gly, Met, Ser, Thr, and Val (Table S5). Notably, mutants with Arg and Trp substitutions in SO068 failed to grow in the fermentation medium containing D-glucose under anaerobic conditions. The effect of amino acid substitutions on succinate yield varied, ranked from highest to lowest as Ser > Val > Ile > Cys > Asn > Gly > Ala in SO068 and Gln > Met > Val > Asp > Thr > Gly > Cys > Ser > Ala in SO069 (Fig. 4). These findings confirm that alterations at F340 in GatC influence sugar specificity and glucose uptake rates.

To further investigate the functional impact of these mutations, CRISPR-Cas9-engineered *gatC* Phe^340^ missense mutant strains in the different genetic backgrounds (Δ*ptsGHI* Δ*exuTR*, and Δ*ptsG* Δ*manX*, Δ*exuTR* Δ*malIXY*) were cultured anaerobically in the fermentation medium with galactitol as the sole carbon source. While Δ*ptsGHI* strains failed to utilize galactitol, Δ*ptsG* strains metabolized galactitol within 24 h (Table S6). This result suggests that galactitol transport remained PTS-dependent regardless of *gatC* mutation. In contrast, the ability of mutant GatC to transport glucose suggests an alternative, non-PTS-mediated uptake mechanism (Fig. S2C).

## Discussion

Deletion of the glucose-specific PTS prevented glucose uptake, leading to impaired cell growth under anaerobic conditions. Through experimental evolution, we obtained adapted progeny strains capable of glucose transport via alternative systems, restoring cell growth. Genomic analysis confirmed that glucose uptake was facilitated by the increased expression of MalX and NagE, potential glucose transporters, due to loss-of-function mutations in the transcriptional regulators MalI and NagC, respectively. Additionally, gain-of-function mutations in GatC, a galactitol-specific transporter, enabled glucose transport.

The glucose-specific PTS normally converts PEP (phosphate donor) to pyruvate while facilitating glucose phosphorylation and transport into the cytoplasm. In its absence, alternative PTS transporters such as the mannose PTS, maltose PTS, and N-acetylglucosamine PTS can transport glucose [20, 21]. GalP has also been reported to transport glucose [22, 23]. MalX and NagE share high sequence similarity with PtsG (52.0% and 45.7%, respectively; https://www.bioinformatics.org/sms2/ident_sim.html) and are potential candidates for glucose transport [24, 25]. Our study confirmed that inactivation of MalI and NagC increased MalX and NagE expression, enabling glucose transport. This is further supported by the observation that deleting MalX and NagE inhibited glucose uptake, resulting in a lag phase (Fig. 1B and S1).

Sugar transport significantly influences carbon flux and metabolite distribution [26]. Our findings demonstrate that anaerobic fermentation profiles vary based on the glucose transport system. Under anaerobic conditions, mixed-acid fermentation produces acetate, formate, lactate, succinate, and ethanol [27, 28]. The glucose-specific PTS is the primary glucose transporter under such conditions, typically yielding only small amounts of succinate. Previously, we reported that glucose transport via ExuT, a non-PTS glucose transporter, abolished lactate production while increasing succinate accumulation under anaerobic conditions, likely due to a reduced intracellular pyruvate pool, which is closely linked to succinate biosynthesis [29]. Interestingly, despite being PTS transporters, MalX and NagE facilitated excessive succinate production. These fermentation profiles suggest that when glucose is transported via the maltose PTS and N-acetylglucosamine PTS, these systems do not function as typical PTS transporters that utilize PEP for sugar phosphorylation, thereby preserving PEP as a precursor for succinate production.

Previous studies have demonstrated that mutations in the EIIB and EIIC domains of PTS transporters can alter sugar specificity. Notably, PtsG mutations have been shown to enable the transport of mannitol, ribose, mannose, and glucosamine [30-33]. Similarly, NagE M263K can facilitate glucose transport [12], while mutations such as S140L and N13S in GatC enable xylose uptake [10, 11]. These findings suggest that sugar specificity can be modulated through mutations in permease.

Building on mutation insights from adaptive laboratory evolution, we successfully engineered strains using the CRISPR-Cas9 system. We observed that substitutions at position F340 in GatC influenced succinate production. Specifically, replacing F340 with Gly or Ala in the Δ*ptsGHI* Δ*exuTR* background led to increased lactate production compared to other engineered strains. By integrating adaptive laboratory evolution with CRISPR-Cas9-based mutagenesis, we successfully generated strains with optimized fermentation profiles through transporter engineering (Table S5).

Functional analysis of the galactitol PTS revealed that both the wild-type and mutated GatC proteins could transport galactitol, indicating that the mutations did not alter its specificity for galactitol. However, in the Δ*ptsGHI* background, galactitol transport was impaired, suggesting that the galactitol PTS phosphorylates galactitol. On the other hand, only galactitol PTS containing a mutated GatC protein could transport glucose into the cytoplasm, regardless of the strain’s genetic background. This suggests that the mutated GatC protein allows glucose uptake, likely acting as a transport channel rather than phosphorylating glucose through the PTS (Fig. S2C).

Overall, this study identified alternative glucose transport systems in *E. coli* through adaptive evolution under anaerobic conditions. Whole-genome analysis revealed key mutations underlying this adaptation, while CRISPR-Cas9-mediated site-specific mutagenesis enabled a deeper investigation into sugar specificity and downstream metabolic flux. Our findings enhance the understanding of sugar transport regulation and gene expression in transporters, offering insights into the link between glucose uptake and downstream metabolism. Furthermore, the combined approach of adaptive evolution and CRISPR-Cas9-based mutagenesis demonstrates its utility for identifying and optimizing microbial glucose transport systems for potential biotechnological applications.

## Supplemental Materials

Supplementary data for this paper are available on-line only at http://jmb.or.kr.



## Figures and Tables

**Table 1:** Fermentation profiles of wild-type, parental, and adapted *Escherichia coli* strains.

Strain	Genotype	Fermentation Time^a^ (h)	OD_600_	Metabolite Concentrations (mM)					
				Residual D-Glucose^b^	Acetate	Ethanol	Formate	Lactate	Succinate
BW25113	Wild-type	9	4.0 ± 0.2	ND^c^	36.4 ± 0.2	37.4 ± 0.2	78.1 ± 0.4	12.2 ± 0.1	5.2 ± 0.0
HK1161	Δ*ptsG* Δ*manX* Δ*exuTR*	84	4.2 ± 0.1	ND	41.8 ± 0.1	19.0 ± 0.4	46.5 ± 0.3	ND	36.0 ± 0.2
HK1165	HK1161 *malI* C251-G262 deletion (Δ12bp)	30	3.9 ± 0.1	2.1 ± 0.4	40.4 ± 0.2	17.3 ± 0.2	47.9 ± 0.1	ND	34.4 ± 0.6
SO023	Δ*ptsG* Δ*manX* Δ*exuTR* Δ*malIXY*	108	4.0 ± 0.1	5.3 ± 0.1	45.3 ± 0.2	21.5 ± 0.0	56.4 ± 0.5	ND	33.0 ± 0.1
SO032	SO023 *nagC* (T769A)	36	3.4 ± 0.1	1.7 ± 0.7	41.6 ± 1.5	17.9 ± 1.4	53.5 ± 3.7	ND	31.5 ± 1.1
HK1162	Δ*ptsGHI* Δ*exuTR*	150	2.5 ± 0.2	3.3 ± 5.7	32.6 ± 5.9	2.6 ± 4.5	38.3 ± 9.5	ND	39.3 ± 4.2
HK1181	HK1162 *gatC* (T1019G)	21	3.3 ± 0.2	ND	36.2 ± 0.4	20.5 ± 0.6	62.0 ± 0.9	2.3 ± 0.1	22.0 ± 0.1

^a^Fermentation time (h) when glucose was completely consumed.

^b^Residual D-glucose concentration. Initially, 50 mM glucose was present in the fermentation medium.

^c^ND, not detected.

**Table 2:** Mutations identified in adapted *Escherichia coli* strains.

Parental strain	Progeny strain	Gene	Function	Mutation type	Nucleotide change	Altered protein
HK1161	HK1165*	*malI*	Maltose regulon, transcriptional repressor	Deletion	12 bp (ΔC251-G262)	Partial deletion
	SO070			Substitution	G115T	E39Z, nonsense
	SO071			Substitution	G664T	E222Z, nonsense
	SO072			Deletion	1 bp (ΔG608)	G203A, frameshift (209Z)
	SO073			Substitution	T713A	L238Z, nonsense
	SO074			Substitution	G560A	W187Z, nonsense
	SO075			Insertion	1 bp (T after T223)	S75F, frameshift (104Z)
SO023	SO032*	*nagC*	N-Acetylglucosamine regulon, transcriptional repressor	Substitution	T769A	C257S, missense
	SO034			Substitution	G770A	C257Y, missense
	SO035			Substitution	G776T	C259F, missense
	SO037			Substitution	T1052A	L351Q, missense
HK1162	HK1181*	*gatC*	Galactitol-specific PTS EIIC component	Substitution	T1019G	F340C, missense

*Whole-genome sequencing was performed.
