# Supplementary material for: Adaptive Evolution of GatC, a Component of the Galactitol Phosphotransferase System, for Glucose Transport in Escherichia coli
Source: J Microbiol Biotechnol. 2025 Apr 23;35:e2502021. doi: 10.4014/jmb.2502.02021 (PMC12089948; doi:10.4014/jmb.2502.02021)
Supplement: Supplementary file 1 [file jmb-35-e2502021-supple.pdf]

J. Microbiol. Biotechnol. 2025. 35: e2502021

https://doi.org/10.4014/jmb.2502.02021

Supplementary Tables and Figures

Adaptive Evolution of GatC, a Component of the Galactitol Phosphotransferase System, for Glucose Transport in *Escherichia coli*

Su On Jeong[1], Hyun Ju Kim[2], and Sang Jun Lee[1]*

[1]Department of Systems Biotechnology, and Institute of Microbiomics, Chung-Ang University, Anseong 17546, Republic of Korea.

[2]Biological Resources Research Department, Nakdonggang National Institute of Biological Resources (NNIBR), Sangju 37242, Republic of Korea.

* **Correspondence:** Sang Jun Lee sangjlee@cau.ac.kr

Table S1. Bacterial strains, phage, and plasmids used in this study.

Name	Relevant Genotypes or Characteristics	Reference/Source
Strain		
BW25113	Δ*(araD-araB)567* Δlac*Z4787(::rrnB-3)* λ^- *rph-1* Δ*(rhaD-rhaB)568 hsdR514*	Laboratory stock
DH5α	*fhuA2 lac(del)U169 phoA glnV44 Φ80' lacZ(del)M15 gyrA96 recA1 relA1 endA1 thi-1 hsdR17*	Laboratory stock
JW0662	BW25113 Δ*nagC*::Km^R	Keio collection
JW0665	BW25113 Δ*nagE*::Km^R	Keio collection
JW1612	BW25113 Δ*malI*::Km^R	Keio collection
JW1613	BW25113 Δ*malX*::Km^R	Keio collection
JW1614	BW25113 Δ*malY*::Km^R	Keio collection
JW2076	BW25113 Δ*gatC*::Km^R	Keio collection
HK1059	MG1655, *araBAD*::P_{BAD}-*cas9*-Km^R	[1]
HK1161	BW25113 Δ*ptsG*, Δ*manX*, Δ*exuTR*::Km^R	[2]
HK1165*	HK1161 adapted strain, *malI* (C251–G262 deletion)	This study
HK1166	HK1161 adapted strain, *malI* (C251–G262 deletion)	This study
HK1167	HK1161 adapted strain, *malI* (C251–G262 deletion)	This study
SO070	HK1161 adapted strain, *malI* (G115T substitution)	This study
SO071	HK1161 adapted strain, *malI* (G664T substitution)	This study
SO072	HK1161 adapted strain, *malI* (G608 Δ1bp)	This study
SO073	HK1161 adapted strain, *malI* (T713A substitution)	This study
SO074	HK1161 adapted strain, *malI* (G560A substitution)	This study
SO075	HK1161 adapted strain, *malI* (T after T223 insertion)	This study
HK1317#	BW25113 Δ*ptsG*, Δ*manX*, Δ*exuTR*::FRT (derived from HK1161)	This study
SO005	BW25113 Δ*ptsG*, Δ*manX*, Δ*exuTR,* Δ*malI*::Km^R (derived from HK1317)	This study
SO023	BW25113 Δ*ptsG*, Δ*manX*, Δ*exuTR*, Δ*malIXY*::Km^R (derived from HK1317)	This study
SO032*	SO023 adapted strain, *nagC* (T769A substitution)	This study
SO034	SO023 adapted strain, *nagC* (G770A substitution)	This study
SO035	SO023 adapted strain, *nagC* (G776T substitution)	This study
SO037	SO023 adapted strain, *nagC* (T1052A substitution)	This study
SO039	BW25113 Δ*ptsG*, Δ*manX*, Δ*exuTR*, Δ*malIXY*::FRT (derived from SO023)	This study
SO048	BW25113 Δ*ptsG*, Δ*manX*, Δ*exuTR*, Δ*malIXY*, Δ*nagC*::Km^R (derived from SO039)	This study
SO057	BW25113 Δ*ptsG*, Δ*manX*, Δ*exuTR*, Δ*malIXY*, Δ*nagC*::FRT (derived from SO048)	This study
SO062	BW25113 Δ*ptsG*, Δ*manX*, Δ*exuTR*, Δ*malIXY*, Δ*nagC*, Δ*nagE*::Km^R (derived from SO057)	This study
HK1162	BW25113 Δ*ptsGHI*, Δ*exuTR*::Km^R	[2]
HK1181*	BW25113 Δ*ptsGHI*, Δ*exuTR*::Km^R, *gatC* (T1019G substitution)	This study
HK1319	BW25113 Δ*ptsGHI*, Δ*exuTR*::FRT (derived from	This study

	HK1162)	
SO068	BW25113 Δ*ptsGHI*, Δ*exuTR, araBAD*::P_{BAD}-*cas9*-Km^R (derived from HK1319)	This study
SO069	BW25113 Δ*ptsG*, Δ*manX*, Δ*exuTR*, Δ*malIXY, araBAD*::P_{BAD}-*cas9*-Km^R (derived from SO039)	This study
Phage		
P1 *vir*	*vir* mutation	S. Adhya (NIH)
Plasmid		
pCP20	pSC101 ori^{ts}, λ repressor, FLP gene, Amp^R, Cm^R	Laboratory stock
pKD46	pSC101 ori^{ts}, *araC*, λ *red* genes, Amp^R	Laboratory stock
pHK463	pSC101 ori^{ts}, *araC*, λ *bet* gene, Amp^R	[1]
pHL003	pSC101 ori^{ts}, Sp^R, Amp^R, sgRNA target (^{498}AGGCTGTCACTGCGGGATCA^{517} in *galK*)	[1]
pSO004	pSC101 ori^{ts}, Sp^R, sgRNA target (^{1007}CCACCATCGGCTTCTTCG^{1024} in *gatC*)	This study

*Whole-genome sequencing was performed.

#FRT indicates that the Km^R marker was deleted, leaving only the FRT sequence.

Table S2. Primers and mutagenic oligonucleotides used in this study.

Name	Sequence (5′→3′)	Description
Primer		
malI-F	GAACAATCTGGATTCAGTAAATTGCGCG	Strain construction
malI-R	GTTGTCAGAATTAAACACATAAACCTCC	
malX-500up	AGCTGCTCACCGTCTTTACCGCCGTG	
malY-500dn	GCACACCAAAGGTGCGGCAACCTTCA	
KmR-half-F	CCAAGCGAAACATCGCATCGAGCGAGCACG	
KmR-half-R	CGATGCGATGTTTCGCTTGGTGGTCGAATG	
malI-500up	GCAACAAGTTGTGACAGATGGAAAAA	
malI-KmR-F	GCAGATAAAGAGAGGAATTCCGGGGATCCGTCGACCTGCAG	
malI-KmR-R	GACGGATCCCCGGAATTCCTCTCTTTATCTGCTATACCTGG	
nagC-500up	ACCCATCTGTACAACGCGATGCCGTA	
nagC-500dn	GCACACAACGCGCCACAAGCGGGATA	
malX-600up	TTCTTCTGCCATCCGTCGCAGGTCAT	
malI-600up	TTTAGATGATGCATAGGAAGTCATGT	
nagC-600up	CAGCAAACTGGCAAATGCCGGGATTG	
nagE-500up	CAACGTGCTTAAAGCTGACCTGGCCT	
nagE-500dn	ATCAGTTCGATCGCATAGGCGTGGAG	
KmR-ATGout	ACCTGCGTGCAATCCATCTTGTTCAATCAT	
16s-rRNA-RTF	CAGCAGCCGCGGTAATAC	RT-qPCR
16s-rRNA-RTR	ACCAGGGTATCTAATCCTGT	
malX-RTF	ATGACGGCGAAAACAGCAC	
malX-RTR	AAAGCAAACGAGCCAATCTTAC	
malY-RTF	TTCCACCCAGCATTACACC	
malY-RTR	GCAAAACCAACCATCAGCC	
nagE-RTF	GGTGACCATCAACCCAGAAA	
nagE-RTR	CAGGAAGTCCGGCAGTTTAATA	
nagB-RTF	TCGCCATATCGTCAATCGTATC	
nagB-RTR	CTGGCCTGCTTTATGCATTTC	
gatC-1005-18F	GTCCACCATCGGCTTCTTCGGTTTTAGAGCTAGAAATAGCA	sgRNA plasmid construction
gatC-1005-18R	ACCGAAGAAGCCGATGGTGGACTAGTATTATACCTAGGACT	
Sm-ATGout	GATACTGGGCCGGCAGGCGCTCCATTGCCC	
Sm-TAAout	GCAATGGAGCGCCTGCCGGCCCAGTATCAG	
pHLts-seq	GCTGTTCAGCAGTTCCTGCCCTCTG	
Mutagenic Oligonucleotide		
gatC_F340X	TTCCACGATGCACGGCGACCGCCATCGCCACGAANNNGCCGATGGTGGCAAGATCGCCAAACGGCAGCAC	Random codon was underlined

Table S3. Fermentation profiles of adapted progeny (*malI* mutation) strains derived from the parental strain HK1161 (Δ*ptsG* Δ*manX* Δ*exuTR*).

Strain	Genotype	Fermentation Time[a] (h)	OD_{600}	Metabolite Concentrations (mM)					
				Residual D-Glucose[b]	Acetate	Ethanol	Formate	Lactate	Succinate
HK1165	HK1161 *malI* C251-262 del (Δ12bp)	30	3.9 ± 0.1	2.1 ± 0.4	40.4 ± 0.2	17.3 ± 0.2	47.9 ± 0.1	ND	34.4 ± 0.6
HK1166	HK1161 *malI* C251-262 del (Δ12bp)	30	3.9 ± 0.3	2.4 ± 0.6	40.2 ± 0.0	17.5 ± 0.1	47.9 ± 0.1	ND	34.0 ± 0.3
HK1167	HK1161 *malI* C251-262 del (Δ12bp)	30	3.7 ± 0.2	2.2 ± 0.2	40.5 ± 0.2	17.6 ± 0.3	49.2 ± 0.2	ND	33.9 ± 0.6
SO070	HK1161 *malI* (G115T)	30	3.4 ± 0.1	ND[c]	38.1 ± 0.4	14.5 ± 0.2	53.2 ± 0.3	ND	34.5 ± 0.5
SO071	HK1161 *malI* (G664T)	30	3.6 ± 0.2	ND	37.9 ± 0.4	14.6 ± 0.2	52.2 ± 1.6	ND	34.8 ± 0.6
SO072	HK1161 *malI* G608 del (Δ1bp)	30	3.5 ± 0.1	ND	38.3 ± 0.1	14.5 ± 0.1	53.4 ± 0.2	ND	34.5 ± 0.2
SO073	HK1161 *malI* (T713A)	30	4.1 ± 0.7	ND	35.9 ± 2.3	19.5 ± 4.6	45.9 ± 6.2	ND	32.9 ± 2.2
SO074	HK1161 *malI* (G560A)	30	3.5 ± 0.1	ND	38.4 ± 0.2	14.4 ± 0.1	53.8 ± 0.3	ND	34.5 ± 0.3
SO075	HK1161 *malI* T after T223 (1bp insertion)	30	3.5 ± 0.2	ND	38.5 ± 0.1	14.9 ± 0.1	54.7 ± 0.3	ND	34.0 ± 0.1
SO005	HK1161 Δ*malI*	30	3.8 ± 0.1	0.5 ± 0.1	42.2 ± 0.1	15.5 ± 0.3	42.8 ± 0.9	ND	32.2 ± 0.7

[a]Fermentation time (h) when glucose was completely consumed.

[b]Residual D-glucose concentration. Initially, 50 mM glucose was present in the fermentation medium.

[c]ND, not detected.

Table S4. Fermentation profiles of adapted progeny (*nagC* mutation) strains derived from the parental strain SO023 (Δ*ptsG* Δ*manX* Δ*exuTR* Δ*malIXY*).

Strain	Genotype	Fermentation Time[a] (h)	OD_{600}	Metabolite Concentrations (mM)					
				Residual D-Glucose[b]	Acetate	Ethanol	Formate	Lactate	Succinate
SO032	SO023 *nagC* (T769A)	36	3.4 ± 0.1	1.7 ± 0.7	41.6 ± 1.5	17.9 ± 1.4	53.5 ± 3.7	ND[c]	31.5 ± 1.1
SO034	SO023 *nagC* (G770A)	36	3.3 ± 0.2	1.2 ± 1.1	42.3 ± 0.8	16.2 ± 0.1	56.1 ± 1.0	ND	30.8 ± 0.7
SO035	SO023 *nagC* (G776T)	36	3.3 ± 0.1	3.3 ± 1.5	44.2 ± 1.4	16.6 ± 0.6	58.3 ± 1.9	ND	32.1 ± 0.9
SO037	SO023 *nagC* (T1052A)	36	3.3 ± 0.1	2.3 ± 1.8	45.1 ± 1.3	19.1 ± 0.7	62.8 ± 2.0	1.3 ± 0.1	28.9 ± 0.7
SO048	SO023 Δ*nagC*	24	3.9 ± 0.1	1.3 ± 0.4	41.8 ± 1.1	23.0 ± 0.6	71.4 ± 2.6	ND	24.4 ± 0.9

[a]Fermentation time (h) when glucose was completely consumed.

[b]Residual D-glucose concentration. Initally, 50 mM glucose was present in the fermentation medium.

[c]ND, not detected.

Table S5. D-glucose fermentation profiles of *gatC* Phe^{340} missense mutant strains at 24 h.

Background Genotype	Missense Mutation	OD_{600}	Metabolite Concentrations (mM)					
			Residual D-Glucose[a]	Acetate	Ethanol	Formate	Lactate	Succinate
SO068 (Δ*ptsGHI* Δ*exuTR* P_{BAD}-*cas9*-Km^R)	Ala	5.1 ± 0.2	ND[b]	31.1 ± 0.1	24.5 ± 0.5	68.5 ± 0.7	31.5 ± 0.2	6.2 ± 0.3
	Arg	0.1 ± 0.0	48.1 ± 0.8	ND	ND	ND	ND	2.0 ± 0.0
	Asn	4.0 ± 0.2	ND	39.6 ± 0.3	28.4 ± 0.1	73.9 ± 0.3	1.7 ± 0.1	17.8 ± 0.8
	Cys	3.9 ± 0.1	ND	40.1 ± 0.2	29.5 ± 0.4	72.4 ± 0.3	2.1 ± 0.2	18.0 ± 0.3
	Gly	2.9 ± 0.0	ND	35.2 ± 0.1	28.9 ± 0.2	57.7 ± 0.3	14.3 ± 0.1	13.9 ± 0.2
	Ile	2.9 ± 0.1	15.3 ± 0.3	28.6 ± 0.6	14.5 ± 0.9	54.1 ± 1.4	ND	13.4 ± 0.1
	Ser	4.1 ± 0.2	ND	41.0 ± 0.2	26.4 ± 0.7	72.7 ± 0.2	1.8 ± 0.2	20.4 ± 0.6
	Trp	0.2 ± 0.0	48.3 ± 0.4	ND	ND	ND	ND	2.1 ± 0.1
	Val	3.2 ± 0.1	7.0 ± 1.0	35.9 ± 0.7	17.9 ± 0.5	69.9 ± 1.5	ND	16.6 ± 0.5
SO069 (Δ*ptsG* Δ*manX* Δ*exuTR* Δ*malIXY* P_{BAD}-*cas9*-Km^R)	Ala	3.7 ± 0.1	ND	40.5 ± 0.1	34.8 ± 0.3	73.4 ± 0.4	2.2 ± 0.3	12.9 ± 0.4
	Asn	4.1 ± 0.6	ND	40.7 ± 0.2	28.3 ± 0.1	70.0 ± 0.2	ND	21.1 ± 0.3
	Cys	4.0 ± 0.3	ND	39.1 ± 0.7	32.2 ± 0.4	60.9 ± 1.3	ND	17.7 ± 0.4
	Gln	4.1 ± 0.1	6.3 ± 0.5	36.1 ± 0.3	13.8 ± 0.3	50.6 ± 0.9	ND	31.1 ± 0.2
	Gly	2.0 ± 0.4	ND	39.3 ± 0.2	30.1 ± 0.1	53.7 ± 0.6	ND	19.8 ± 0.4
	Met	4.8 ± 0.0	ND	35.8 ± 0.8	21.1 ± 0.6	48.0 ± 1.2	ND	30.7 ± 0.7
	Ser	3.4 ± 0.4	ND	41.1 ± 0.3	33.5 ± 0.6	74.5 ± 1.0	1.7 ± 0.2	14.2 ± 0.6
	Thr	4.5 ± 0.1	ND	38.6 ± 0.1	29.8 ± 0.2	57.9 ± 0.4	ND	20.2 ± 0.3
	Val	3.9 ± 0.2	ND	36.2 ± 0.0	24.4 ± 0.1	47.1 ± 0.6	ND	26.3 ± 0.2

[a]Residual D-glucose concentration. Initally, 50 mM glucose was present in the fermentation medium.

[b]ND, not detected.

Table S6. Galactitol fermentation profiles of *gatC* Phe[340] missense mutant strains at 24 h.

Background Genotype	Missense Mutation	OD_{600}	Metabolite Concentrations (mM)					
			Residual Galactitol[a]	Acetate	Ethanol	Formate	Lactate	Succinate
SO068 (Δ*ptsGHI* Δ*exuTR* P_{BAD}-*cas9*-Km[R])	Ala	0.2 ± 0.0	48.9 ± 0.1	ND	ND	ND	ND	1.1 ± 0.0
	Arg	0.1 ± 0.0	46.3 ± 0.2	ND	ND	ND	ND	1.9 ± 0.0
	Asn	0.2 ± 0.1	48.6 ± 0.0	ND	ND	ND	ND	2.0 ± 0.0
	Cys	0.2 ± 0.0	48.8 ± 0.2	ND	ND	ND	ND	1.9 ± 0.0
	Gly	0.2 ± 0.0	48.8 ± 0.3	ND	ND	ND	ND	2.0 ± 0.0
	Ile	0.2 ± 0.1	47.1 ± 0.3	ND	ND	ND	ND	2.0 ± 0.0
	Ser	0.2 ± 0.0	48.9 ± 0.1	ND	ND	ND	ND	2.0 ± 0.0
	Trp	0.2 ± 0.0	46.8 ± 0.4	ND	ND	ND	ND	2.0 ± 0.0
	Val	0.2 ± 0.0	47.9 ± 0.6	ND	ND	ND	ND	2.1 ± 0.0
SO069 (Δ*ptsG* Δ*manX* Δ*exuTR* Δ*malIXY* P_{BAD}-*cas9*-Km[R])	Ala	3.6 ± 0.2	ND	21.4 ± 0.1	61.2 ± 0.1	93.3 ± 0.3	ND	8.5 ± 0.0
	Asn	3.6 ± 0.1	ND	22.2 ± 0.3	60.4 ± 0.2	92.4 ± 1.2	ND	9.5 ± 0.1
	Cys	3.9 ± 0.1	ND	21.1 ± 1.0	61.4 ± 0.2	88.7 ± 1.9	ND	7.6 ± 0.1
	Gln	3.3 ± 0.0	ND	21.3 ± 0.9	60.7 ± 0.3	89.3 ± 1.3	ND	9.9 ± 0.2
	Gly	3.1 ± 0.0	ND	20.8 ± 0.9	59.6 ± 0.4	86.5 ± 1.9	ND	10.4 ± 0.4
	Met	3.8 ± 0.1	ND	21.1 ± 1.1	60.9 ± 0.9	89.3 ± 2.1	ND	7.6 ± 0.1
	Ser	3.4 ± 0.1	ND	23.4 ± 0.6	59.8 ± 0.4	99.6 ± 1.9	ND	9.1 ± 0.1
	Thr	3.7 ± 0.2	ND	21.7 ± 0.9	61.3 ± 0.1	89.9 ± 1.8	ND	8.3 ± 0.1
	Val	3.4 ± 0.1	ND	20.9 ± 1.0	60.2 ± 0.3	88.6 ± 2.0	ND	9.8 ± 0.2

[a]Residual galactitol concentration. Initially, 50 mM galactitol was present in the fermentation medium.

[b]ND, not detected.

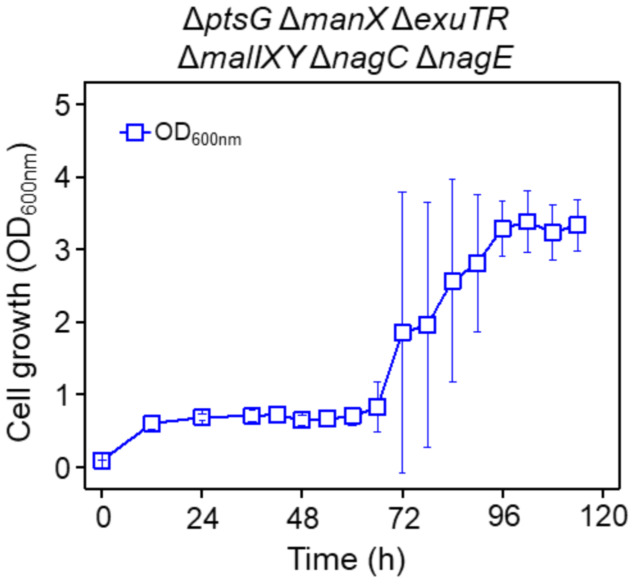

Fig. S1. Prolonged growth of the SO062 strain deficient in alternative glucose transporters (Δ*ptsG* Δ*manX* Δ*exuTR* Δ*malIXY* Δ*nagC* Δ*nagE*).

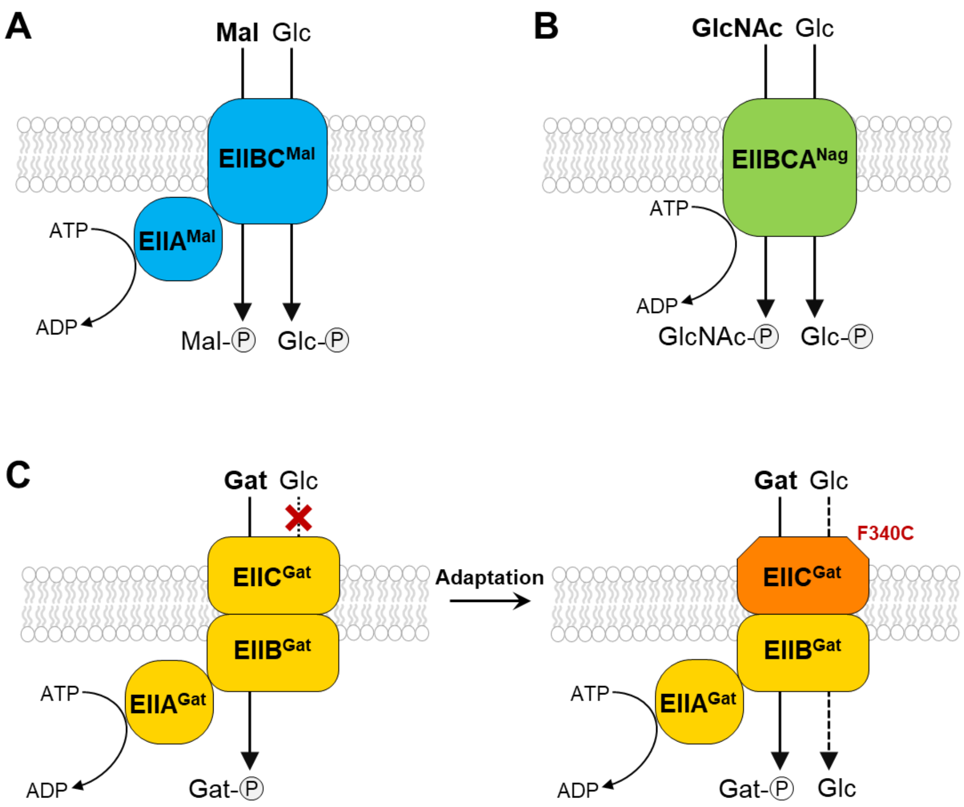

Fig. S2. Adaptation of alternative glucose transport systems in *Escherichia coli*. (A) Maltose phosphotransferase system (PTS), (B) N-acetylglucosamine PTS, and (C) galactitol PTS function as alternative glucose transporters in adapted progeny strains. These systems facilitate efficient glucose uptake and utilization under anaerobic conditions.

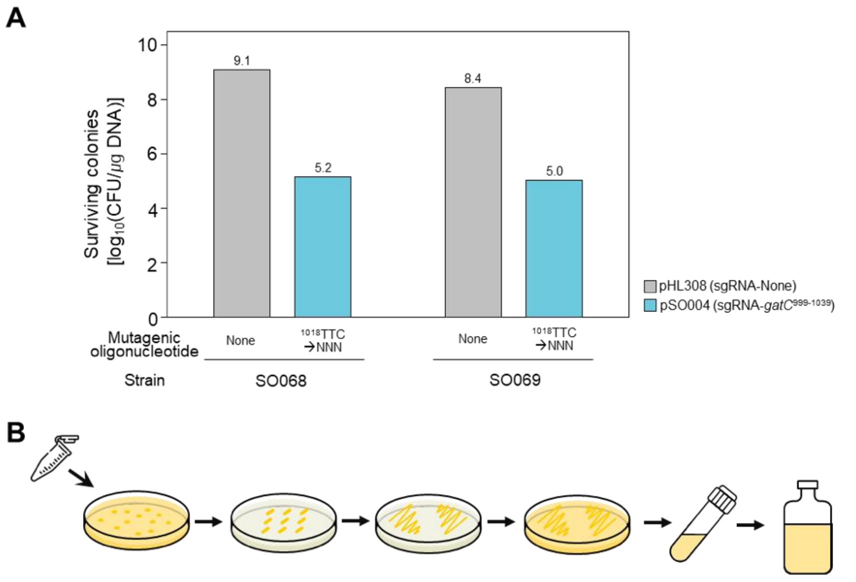

	LB+Spectinomycin (aerobic)	Fermentation medium (anaerobic) 1'	2'	LB medium (aerobic)	Fermentation (anaerobic)
SO068	66	22	18	13	13
SO069	66	30	30	25	25

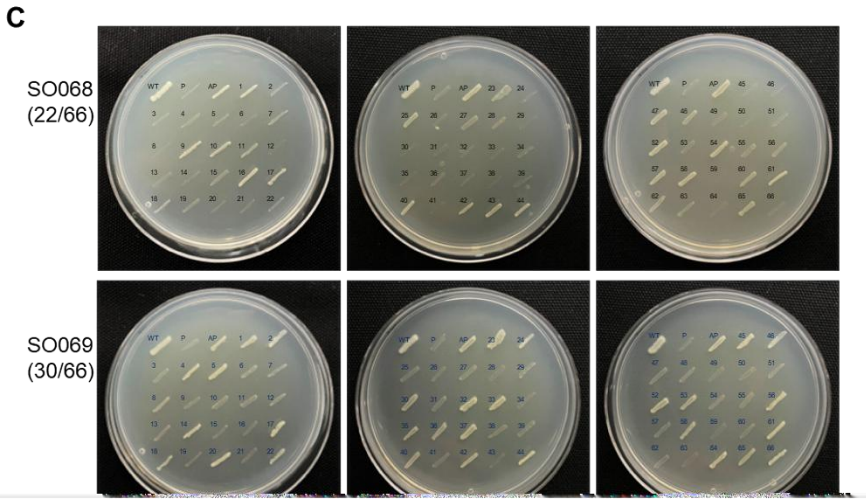

Fig. S3. CRISPR-Cas9 mediated site-directed random mutagenesis and screening process of the *gatC* gene. (A) Reduction in surviving colonies due to negative selection. pHL308 is a non-target sgRNA. When *gatC*-targeting sgRNA (pSO004) and a mutagenic oligonucleotide are transformed, the number of surviving colonies decreases due to negative selection. (B) Selection and purification of mutants carrying random codon insertions in the *gatC* gene. (C) Screening results of primary transformants in fermentation medium under anaerobic conditions.

Supplementary Methods

Strain Construction

To generate a kanamycin-sensitive strain, HK1161 and HK1165 (HK1161 *malI* C251-262 del, Δ12bp) were transformed with the pCP20 plasmid to remove the kanamycin resistance cassette via FLP recombinase-mediated excision. Transformed cells carrying pCP20 were plated on LB agar containing ampicillin (50 μg mL^{-1}) and incubated at 30°C. Single colonies were isolated and incubated at 42°C to cure the pCP20 plasmid.

To generate SO005 and SO015 (Δ*ptsG* Δ*manX* Δ*exuTR* Δ*malI*), P1 *vir* phage lysates from BW25113 Δ*malI*::Km^R (JW1612) were used to transduce HK1161 and HK1165.

To generate Δ*malXY* and Δ*malIXY* strains, a *malXY*::Km^R cassette was constructed via overlap PCR. The first DNA fragment, encompassing the 500-bp upstream region of *malX* and the first 853 bp of the Km^R cassette, was amplified using Δ*malX*::Km^R (JW1613) as a template with primers malX-500up (5′-AGCTGCTCACCGTCTTTACCGCCGTG-3′), and KmR-half-R (5′-CGATGCGATGTTTCGCTTGGTGGTCGAATG-3′). The second DNA fragment, containing the last 470 bp of the Km^R cassette and the 500-bp downstream region of *malY* was amplified using Δ*malY*::Km^R (JW1614) as a template with primers KmR-half-F (5′-CCAAGCGAAACATCGCATCGAGCGAGCACG) and malY_500dn (5′-GCACACCAAAGGTGCGGCAACCTTCA). These fragments were fused via overlap PCR to construct Δ*malXY*::Km^R, which was electroporated into L-arabinose-induced HK1317 and SO014 (kanamycin-sensitive derivatives of HK1161 and HK1165) to generate SO017 (Δ*ptsG* Δ*manX* Δ*exuTR* Δ*malXY*) and SO018 (Δ*ptsG* Δ*manX* Δ*exuTR* Δ*malXY* *malI* C251-262 deletion), respectively.

The Δ*malIXY*::Km^R cassette was also generated via overlap PCR. The first DNA fragment, comprising the 500-bp upstream region of *malI* and a 16-bp overhang sequence of the Km^R cassette (16 bp), was amplified using Δ*malI*::Km^R (JW1612) as a template with primers malI-

500up (5′-GCAACAAGTTGTGACAGATGGAAAAA-3′) and malI-KmR-R (5′-GACGGATCCCCGGAATTCCTCTCTTTATCTGCTATACCTGG-3′). The second DNA fragment, containing the 16-bp overhang of *malI*, the Km^R cassette, and the 500-bp downstream region of *malY* gene, was amplified using Δ*malY*::Km^R (JW1614) as a template with primers malI-KmR-F (5′-GCAGATAAAGAGAGGAATTCCGGGGATCCGTCGACCTGCAG-3′) and malY_500dn (5′-GCACACCAAAGGTGCGGCAACCTTCA-3′). These fragments were fused via overlap PCR and electroporated into L-arabinose-induced SO005 and SO015 to generate SO023 and SO024 (Δ*ptsG* Δ*manX* Δ*exuTR* Δ*malIXY*), respectively.

Adapted strains of HK1162 (HK1181) and SO023 (SO032, SO034, SO035, and SO037) were isolated as described above. To delete the *nagC* gene in SO023 (parental strain) and SO032 (adapted strain), the Km^R cassette was deleted using FLP recombinase, generating SO039 and SO040, respectively. The *nagC*::Km^R cassette was amplified from Δ*nagC*::Km^R (JW0662) with primers nagC-500up (5′- ACCCATCTGTACAACGCGATGCCGTA-3′) and nagC-500dn (5′-GCACACAACGCGCCACAAGCGGGATA-3′). The purified PCR products were electroporated into L-arabinose-induced SO039 and SO040, yielding SO048 and SO049 (Δ*ptsG* Δ*manX* Δ*exuTR* Δ*malIXY* Δ*nagC*), respectively.

To delete *nagE*, the *nagE*::Km^R cassette was amplified using Δ*nagE*::Km^R (JW0665) as a template with primers of nagE-500up (5′-CAACGTGCTTAAAGCTGACCTGGCCT-3′) and nagE-500dn (5′-ATCAGTTCGATCGCATAGGCGTGGAG-3′). The purified PCR products were electroporated into L-arabinose-induced SO048 and SO049, generating SO062 and SO063 (Δ*ptsG* Δ*manX* Δ*exuTR* Δ*malIXY* Δ*nagC* Δ*nagE*), respectively.

Genome Analysis

Genomic DNA (100 ng) was fragmented using adaptive focused acoustic technology (Covaris). This fragmented DNA was subjected to end repair to generate blunt-ended, 5'-phosphorylated double-stranded DNA molecules, followed by bead-based size selection. A single 'A' base was added to each fragment, and TruSeq DNA UD Indexing adapters (Illumina, Inc., San Diego, CA, USA) were ligated. The final library was amplified via PCR and quantified using qPCR (KAPA Library Quantification Kit for Illumina). Library quality and fragment size distribution were assessed using the Agilent 4200 TapeStation (D1000 ScreenTape; Agilent Technologies, Santa Clara, California, USA). Finally, sequencing was performed on the Illumina NovaSeq platform.

Post-sequencing, raw reads were retrieved from the NCBI Sequence Read Archive (SRA) and processed using Trimmomatic (v0.38) [3] to remove adapter sequences and low-quality reads, minimizing analysis bias. Read quality was evaluated using FastQC (v0.11.8) (https://www.bioinformatics.babraham.ac.uk/projects/fastqc/). High-quality reads were mapped to the *E. coli* BW25113 reference genome (NCBI RefSeq, GeneBank: CP009273.1, https://www.ncbi.nlm.nih.gov/nuccore/CP009273) using BWA-mem (v0.7.17) [4]. Duplicate reads were removed using Sambamba (v0.6.8) [5].

Genome coverage and mapping ratios were calculated, and variant calling was performed using SAMtools (v.1.9) [6] and BCFtools (v.1.9) (http://samtools.github.io/bcftools/call-m.pdf). SNPs and short indels with a Phred score > 30 (≥ 99.9% base call accuracy) were identified and annotated using SnpEff (v.4.3t) [7]. SnpEff databases, generated from the General Feature Format (gff) file of the reference genome, enabled prediction of annotation types, putative impacts, and amino acid changes.

References

1. Lee HJ, Kim HJ, Lee SJ. 2020. CRISPR-Cas9-mediated pinpoint microbial genome editing aided by target-mismatched sgRNAs. *Genome Res.* **30:** 768-775.
2. Kim HJ, Jeong H, Lee SJ. 2020. Short-Term Adaptation Modulates Anaerobic Metabolic Flux to Succinate by Activating ExuT, a Novel D-Glucose Transporter in *Escherichia coli*. *Front. Microbiol.* **11:** 27.
3. Bolger AM, Lohse M, Usadel B. 2014. Trimmomatic: a flexible trimmer for Illumina sequence data. *Bioinformatics.* **30:** 2114-2120.
4. Li H, Durbin R. 2010. Fast and accurate long-read alignment with Burrows-Wheeler transform. *Bioinformatics.* **26:** 589-595.
5. Tarasov A, Vilella AJ, Cuppen E, Nijman IJ, Prins P. 2015. Sambamba: fast processing of NGS alignment formats. *Bioinformatics.* **31:** 2032-2034.
6. Li H. 2011. A statistical framework for SNP calling, mutation discovery, association mapping and population genetical parameter estimation from sequencing data. *Bioinformatics.* **27:** 2987-2993.
7. Cingolani P, Platts A, Wang le L, Coon M, Nguyen T, Wang L*, et al.* 2012. A program for annotating and predicting the effects of single nucleotide polymorphisms, SnpEff: SNPs in the genome of Drosophila melanogaster strain w1118; iso-2; iso-3. *Fly (Austin).* **6:** 80-92.
